# Combinatorial Analysis of Phenotypic and Clinical Risk Factors Associated With Hospitalized COVID-19 Patients

**DOI:** 10.3389/fdgth.2021.660809

**Published:** 2021-07-08

**Authors:** Sayoni Das, Matthew Pearson, Krystyna Taylor, Veronique Bouchet, Gert Lykke Møller, Taryn O. Hall, Mark Strivens, Kathy T. H. Tzeng, Steve Gardner

**Affiliations:** ^1^PrecisionLife Ltd., Oxford, United Kingdom; ^2^OptumLabs at UnitedHealth Group, Minnetonka, MN, United States

**Keywords:** COVID-19, SARS-CoV-2, severe COVID-19, disease risk, patient stratification, combinatorial analysis, real world data analysis

## Abstract

Characterization of the risk factors associated with variability in the clinical outcomes of COVID-19 is important. Our previous study using genomic data identified a potential role of calcium and lipid homeostasis in severe COVID-19. This study aimed to identify similar combinations of features (disease signatures) associated with severe disease in a separate patient population with purely clinical and phenotypic data. The PrecisionLife combinatorial analytics platform was used to analyze features derived from de-identified health records in the UnitedHealth Group COVID-19 Data Suite. The platform identified and analyzed 836 disease signatures in two cohorts associated with an increased risk of COVID-19 hospitalization. Cohort 1 was formed of cases hospitalized with COVID-19 and a set of controls who developed mild symptoms. Cohort 2 included Cohort 1 individuals for whom additional laboratory test data was available. We found several disease signatures where lower levels of lipids were found co-occurring with lower levels of serum calcium and leukocytes. Many of the low lipid signatures were independent of statin use and 50% of cases with hypocalcemia signatures were reported with vitamin D deficiency. These signatures may be attributed to similar mechanisms linking calcium and lipid signaling where changes in cellular lipid levels during inflammation and infection affect calcium signaling in host cells. This study and our previous genomics analysis demonstrate that combinatorial analysis can identify disease signatures associated with the risk of developing severe COVID-19 separately from genomic or clinical data in different populations. Both studies suggest associations between calcium and lipid signaling in severe COVID-19.

## Introduction

The Coronavirus disease 2019 (COVID-19) outbreak caused by the severe acute respiratory syndrome coronavirus 2 (SARS-CoV-2) has been declared a pandemic that has resulted in significant mortality, major social and economic disruption worldwide (1). The uncertainty surrounding the progression, management, and outcomes of COVID-19 has made it particularly challenging for healthcare systems. Studies have suggested that ~80% of COVID-19 positive patients present with mild symptoms or are asymptomatic and that around 20% of the patients develop a more severe response that may lead to hospitalization and, in some cases (2.3%), death (2–5).

The risk of developing severe COVID-19 is known to be higher in people who are older, male and have underlying health conditions such as hypertension, cardiovascular disease, diabetes, obesity, chronic respiratory diseases, and cancer (4, 5). Approximately 22% of the global population have at least one co-morbidity that puts them at increased risk of severe COVID-19 if exposed to the virus (6). Ethnicity and socio-economic deprivation have also been associated with severe illness (7).

SARS-CoV-2 binds to the host cell receptor through angiotensin-converting enzyme-2 (ACE2) (8) and starts replicating rapidly inside the host cells, which can trigger a hyperimmune response in some patients (9). This may be due to the generation of pro-inflammatory cytokines and chemokines called a cytokine storm that can cause acute respiratory distress syndrome (ARDS) in the lung and multi-organ failure (10, 11). Other studies have suggested that binding of SARS-CoV-2 increases the levels of ACE2 in lung cells that results in elevated levels of bradykinin (12) (bradykinin storm) leading to vascular leakage, hypotension, and pulmonary edema (13). These are manifested in COVID-19 patients with pneumonia and respiratory failure. Bradykinin's role in the regulation of clotting may be one mechanism for the extra-pulmonary manifestations such as thromboembolic complications, cardiac events, acute renal and hepatic injury (14, 15). Other symptoms such as neurological complications and gastrointestinal and endocrine symptoms have also been reported (14, 16). Recent evidence suggests that some patients with COVID-19 can also develop long-term complications or experience prolonged symptoms (17, 18).

Early identification and characterization of the risk factors associated with varying clinical outcomes of severely ill COVID-19 patients are crucial for accurate clinical stratification and the development of effective management and targeted therapeutic strategies. A previous case-control study using genomic data (19) identified 68 severe COVID-19 risk-associated genes in a population of hospitalized COVID-19 patients in the UK Biobank (20, 21). Nine of these were previously linked to differential response to SARS-CoV-2 infection. Several of these genes are related to key biological pathways associated with the development of severe COVID-19 and associated symptoms, including cytokine production cascades, endothelial cell dysfunction, lipid droplets, calcium signaling, and viral susceptibility factors (19).

In this study, we identified and assessed the phenotypic and clinical risk factors associated with hospitalized COVID-19 patients in the UnitedHealth Group (UHG) COVID-19 Data Suite using a similar combinatorial analysis approach. Using laboratory test data available for the UHG cohort, we investigated potential correlations with the genomic analysis findings and hypotheses from our previous UK Biobank COVID-19 study (19), including the potential association of calcium signaling and lipid dysregulation with severe clinical outcomes in COVID-19 patients.

## Method

### Cohort Generation

We used de-identified records of Medicare Advantage and commercially insured members with COVID-19 test results in the UHG COVID-19 Data Suite accessed through the UHG Clinical Discovery Portal for this study. The UHG COVID-19 Data Suite contains longitudinal health information on individuals representing diverse ethnicities, age groups, and geographical regions across the United States. The information includes data on COVID-19 test results, in-patient admission data for hospitalized individuals, medical and pharmacy claims, general diagnostic information, demographic data, and information on healthcare insurance plans.

We performed case-control studies on two cohorts to identify combinatorial disease signatures associated with the risk of hospitalization for COVID-19 positive patients. Cohort 1, consisting of 9,493 individuals (3,183 cases, 6,310 controls), was generated from the UHG COVID-19 Data Suite (dated August 2020). This contained 3,183 cases who had been hospitalized as a result of developing severe COVID-19 (based on primary diagnosis records) and 6,310 mild controls who had tested positive for COVID-19 but not been hospitalized (Supplementary Table 1). Patients who were enrolled in the Medicare Special Needs Plan were excluded to reduce any confounding factors associated with these patients, who are often above 65 years old and diagnosed with severe/disabling chronic conditions that increase their risk of hospitalization. Patients without linked clinical data since 2019 were also excluded.

To investigate the potential role of calcium and lipid homeostasis in COVID-19 patients with severe clinical outcomes, we selected five laboratory analytes that were relevant for this hypothesis and had good coverage in Cohort 1. These included serum calcium, low-density cholesterol (LDL), high-density cholesterol (HDL), triglycerides, and leukocyte count. A subcohort, Cohort 2, consisting of 1,581 patients (581 cases and 1,000 controls) was generated for the individuals with laboratory test results for these five analytes.

### Feature Generation

The clinical, claims and pharmacy data were converted to categorical features for the study (Supplementary Section Feature Generation). The clinical and phenotypic data available for all individuals in Cohort 1 generated 1,339 binary features per patient (Supplementary Table 2). An additional, five laboratory analyte features were added for Cohort 2.

### Combinatorial Analysis

The PrecisionLife platform uses a proprietary data analytics framework that enables efficient combinatorial analysis of large, n-dimensional, multi-modal patient datasets. Navigating this data space allows for the identification of combinations of features that are significantly associated with groups of cases in a case-control dataset.

Traditional analysis methods typically identify single features in a dataset that are important for a relatively large number of cases associated with a disease diagnosis. They may seek to combine these single feature effects using a variety of methods. However, most large disease populations are heterogenous with multiple features coming together to exert non-linear influences on disease biology that lead to patient sub-populations having different symptoms, progression, and/or outcomes. These non-linear effects can only be observed in combination, i.e., they are a product of the interaction and so have to be observed and modeled at that level. The combinatorial approach used in this analysis enables us to capture the non-linear effects of these interactions on a disease (e.g., the effects of feedback loops in metabolic or genetic networks), which can only be seen in combinations found to be significant in such patient subgroups. This approach has been validated in multiple disease populations (19, 22, 23).

PrecisionLife's combinatorial analysis algorithm comprises two main phases: mining and processing (Supplementary Figure 1). In the mining phase, the algorithm identifies and validates combinations of feature states (for example SNP and associated genotype state) that are over-represented in cases. Multiple feature states are combined iteratively (using a Z-score statistic) until no additional single feature state is added. Combinations of feature states that have high odds ratios and high penetrance are prioritized. The mining process is repeated for 2,500 cycles of fully randomized permutation of all individuals in the dataset, keeping the same parameters and case:control ratio.

All combinations associated with each feature state are identified to form 'simple networks' for the original dataset and for each iteration of random permutation of the dataset. The simple networks are then validated using network properties such as minimum penetrance (number of cases in the simple network) as the null hypothesis when compared with the networks of the random permutations. Simple networks that appear in the random permutations above a preset FDR threshold are considered to be random and eliminated. All disease signatures from the validated simple networks are reported as validated disease signatures.

In the last phase, the validated disease signatures are processed. The features that connect all disease signatures in validated simple networks (known as critical features) are identified. These critical features are scored using a Random Forest (RF) algorithm based inside a 5-fold cross-validation framework to evaluate the accuracy with which the feature predicts the observed case:control split (minimizing Gini impurity) in a dataset. We use the resulting score to rank disease signatures.

Finally, a merged network architecture is generated by clustering all validated disease signatures based on their co-occurrence in patients in the dataset.

The PrecisionLife platform generated statistically significant disease signatures containing up to five features for each cohort. Each analysis took less than an hour to complete, running on a 32 CPU, 4 GPU cloud compute server. These were mapped to the cases in which they were found, and in-patient clinical data were used to generate a patient profile for each combinatorial disease signature.

## Results

### Cohort Characteristics

Cohort 1 patients (3,183 cases) had a 19.1% (607 cases) mortality rate, while 51.3% (1,548 cases) were released from care and 29.6% (915 cases) were transferred to other healthcare facilities. Within Cohort 1, 51.3% were female, and 66.7% were Caucasian with a median age of 75 (Table 1, Supplementary Figure 2).

**Table 1:** Cohort characteristics for the hospitalization risk studies.

	**Cohort 1 (*****n*** **= 9,493)**			**Cohort 2 (*****n*** **= 1,581)**		
	**Hospitalized patients**	**Non-hospitalized patients**	**Two-sided *p-value***	**Hospitalized patients**	**Non-hospitalized patients**	**Two-sided *p*-value**
COVID-19 positive	3,183	6,310	N/A	581	1,000	N/A
Males (%)	1,549 (48.7%)	2,758 (43.7%)	5.0e-06	295 (50.1%)	348 (34.8%)	6.5e-10
Median Age (Range)	75 (29–89)	72 (15-89)	<2.2e-16	74 (31–89)	71.5 (33–89)	2.2e-14
Caucasian^*^ (%)	1,632 (66.7%)	3,693 (66.8%)	0.938	298 (57.5%)	528 (59.5%)	0.465
Mortality (%)	607 (19.10%)	N/A	N/A	100 (17.2%)	N/A	N/A
Hypertension (%)	1,657 (52.1%)	3,864 (61.2%)	<2.2e-16	431 (58.7%)	672 (67.2%)	3.7e-03
Diabetes (%)	1,109 (34.8%)	2,114 (33.5%)	0.198	277 (47.7%)	427 (42.7%)	5.8e-02
Cardiovascular (%)	1,210 (38.0%)	1,468 (23.3%)	<2.2e-16	204 (35.1%)	267 (26.7%)	2.2e-03
Dementia (%)	443 (13.9%)	236 (3.7%)	<2.2e-16	27 (4.6%)	10 (1.0%)	7.2e-06
Chronic lung disease (%)	832 (25.9%)	1,198 (20.0%)	2.4e-15	135 (23.2%)	188 (18.9%)	3.8e-02

* *The percentage of Caucasians was calculated as a fraction of those individuals for whom ethnicity information was available (~85% of all records)*.

*p-values were calculated to assess the association of each feature in the two COVID-19 hospitalized risk cohorts using two-sided Fisher's exact tests for categorical data and Mann-Whitney U tests for continuous data*.

Around 54% of the hospitalized patients had at least one of the comorbidities previously linked with higher risk for COVID-19 severe response. Hypertension (52.1%) was the most common co-morbidity, followed by cardiovascular disease (38%), diabetes (31.5%), chronic lung disease (25.9%) and dementia (13.9%) (Table 1). The most common COVID-19 related diagnoses reported in hospital admissions data for cases were pneumonia (43%), followed by respiratory failure (18.3%) and septicemia (7.3%) (Supplementary Figure 3).

### Combinatorial Disease Signatures Capture Phenotypic and Clinical Risk Factors for Severe COVID-19

The combinatorial analysis identified 1,147 combinations of clinical and phenotypic features (disease signatures) that were highly associated with hospitalized patients in Cohort 1 and 32,242 combinations in Cohort 2 (Supplementary Table 3, Supplementary Figure 4). A higher number of disease signatures was reported for Cohort 2. This is likely due to the relatively higher prevalence of the same clinical features among Cohort 2 individuals as compared to Cohort 1.

The disease signatures were filtered to exclude those that had any features indicating an absence of a disease diagnosis, symptom, or medication use, as these are likely to be generated as a result of incompleteness of the claims and pharmacy data rather than as a true disease association. Additionally, disease signatures that were found in fewer than 20 cases were also excluded. After filtering, 255 disease signatures in Cohort 1 and 531 disease signatures in Cohort 2 were used for further analysis.

All features in the disease signatures identified for each study were scored using a Random Forest (RF) algorithm based inside a 5-fold cross-validation framework to evaluate the accuracy with which a feature (e.g., a laboratory analyte value) predicts the observed case:control split (minimizing Gini impurity). One hundred sixty-six features in Cohort 1 and 41 features in Cohort 2 were identified as critical features as shown in Supplementary Figure 5. Many of these included diagnoses and symptoms associated with severe COVID-19 such as respiratory failure, pneumonia, acute renal failure, and septicemia because of their low incidence in controls.

**Figure 1:**
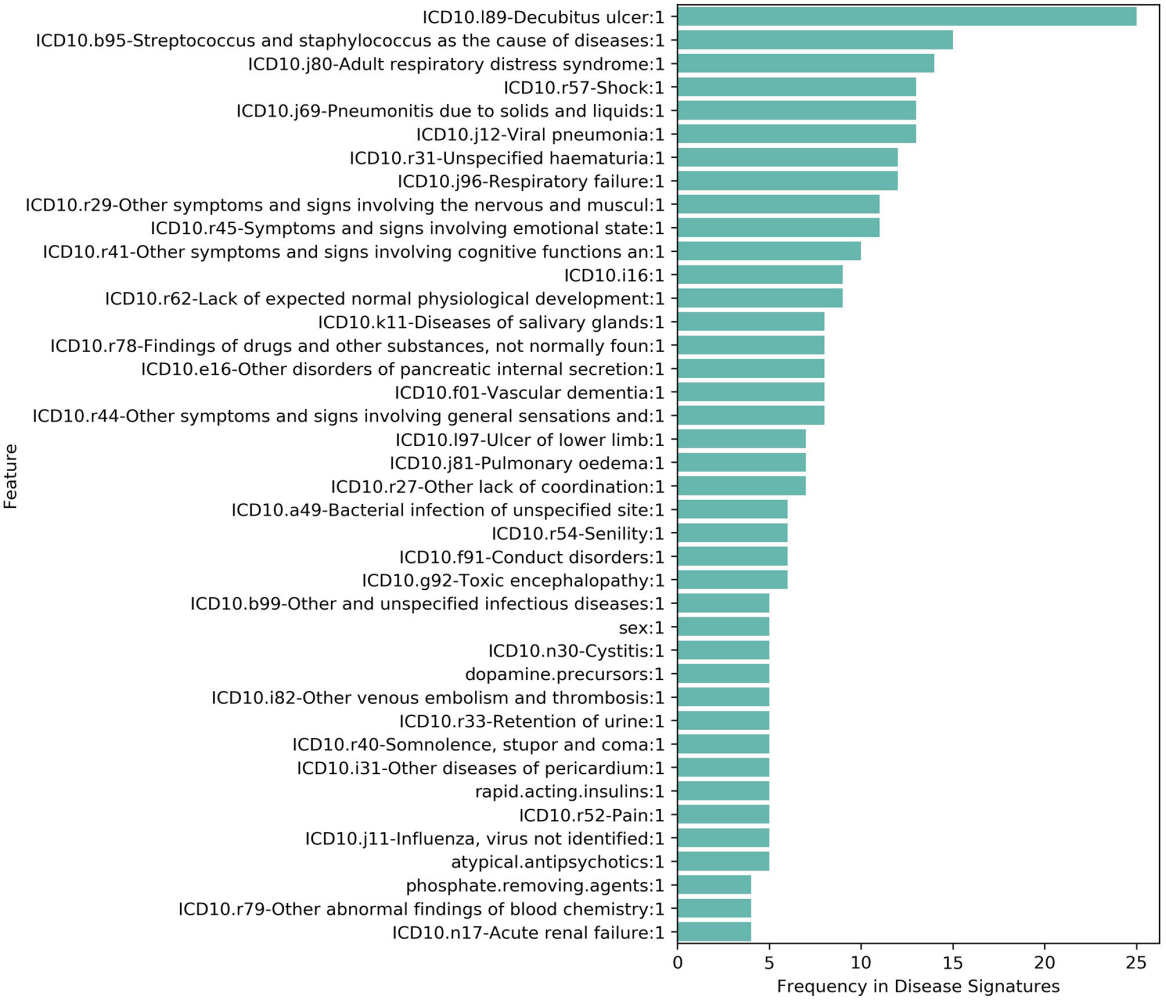
Phenotypic and clinical features that were most frequently reported (top 40) in 255 filtered disease signatures in Cohort 1 were associated with an increased risk of hospitalization with severe COVID-19.

**Figure 2:**
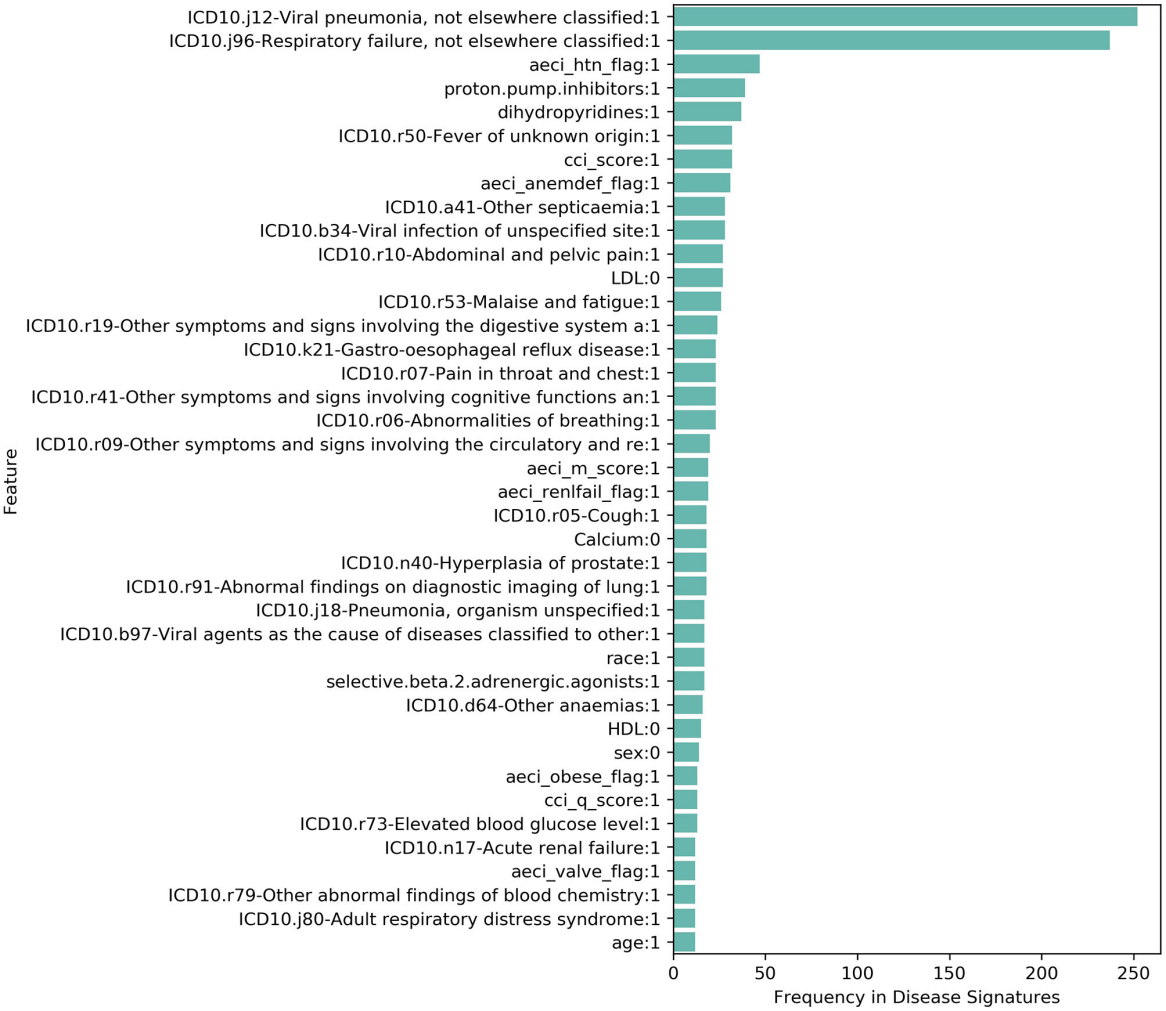
Phenotypic and clinical features that were most frequently reported (top 40) in 581 filtered disease signatures in Cohort 2 (subcohort of Cohort 1 with additional laboratory test results) associated with increased risk of hospitalization with severe COVID-19. Features associated with hypocalcemia (Calcium:0) and hypolipidemia (LDL:0, HDL:0) were reported in multiple disease signatures.

We found that the combinatorial disease signatures capture clinical features associated with response to severe COVID-19 illness (Figures 1, 2) These features include pneumonia and respiratory failure, which are frequently reported among hospitalized patients, and risk factors that increase the probability of developing severe response such as diabetes, hypertension and cardiovascular disease. Phenotypes related to the risk-associated comorbidities such as elevated glucose levels or blood pressure and common medications prescribed for them (e.g., insulin, statins, and dihydropyridines) were also commonly found. Many low-frequency features (<10% among hospitalized patients) such as ARDS (10), pneumothorax (24), hematuria (25), encephalopathy (16), pericarditis (26), and thrombosis (14) were frequently found in disease signatures in combination with other features. Some disease signatures also captured clinical features related to increased frailty such as senility or high risk of hospital readmission, whilst other features reflect conditions that are associated with prolonged hospital stay, such as pressure ulcers and secondary bacterial infections.

**Figure 3:**
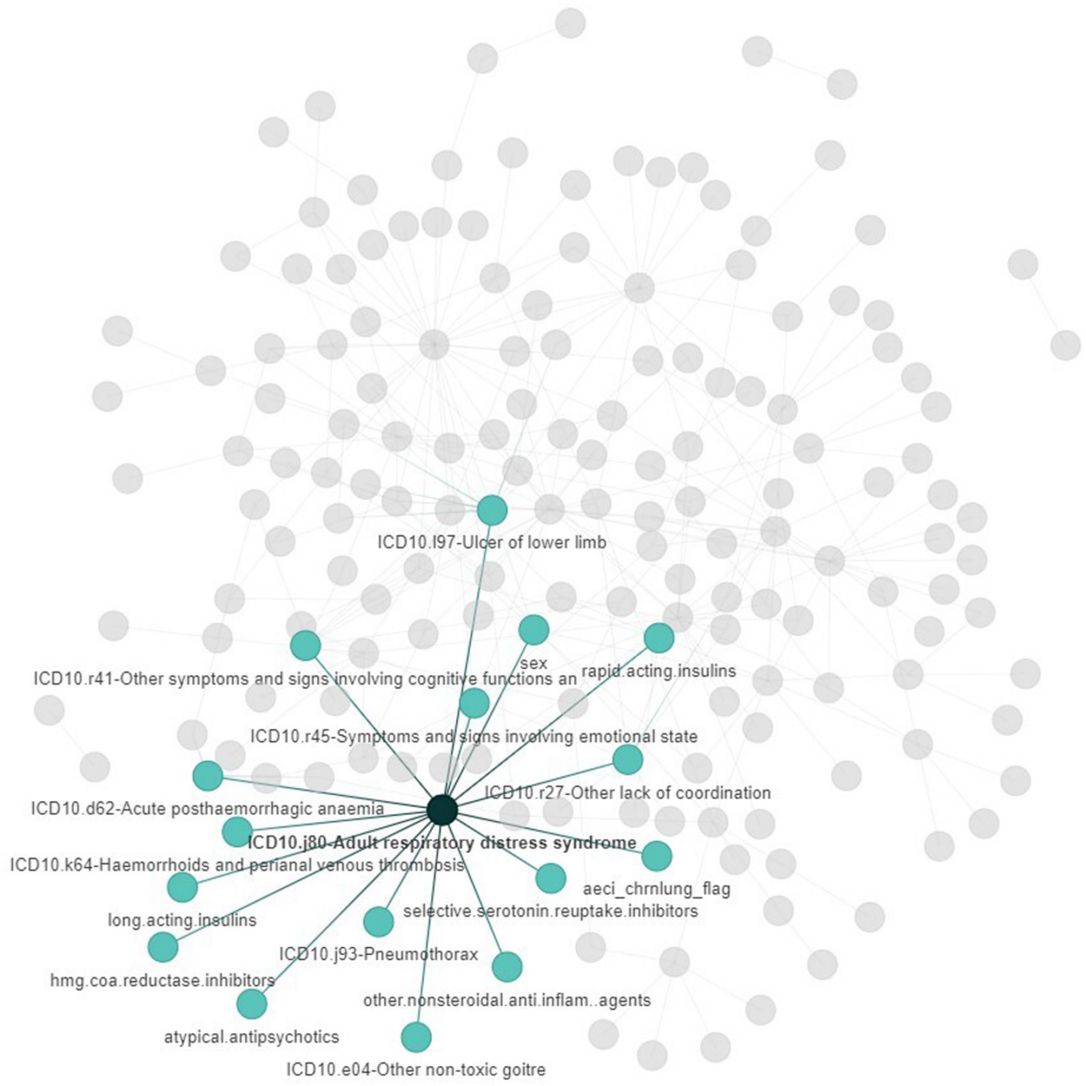
The network architecture of filtered (*n* = 255) disease signatures associated with hospitalized COVID-19 patients in Cohort 1 generated by the PrecisionLife platform where each circle represents a feature and edges represent co-association in patients. The colored nodes and edges represent the disease signatures of patients who developed ARDS (shown in a darker shade) in Cohort 1. The co-associated features are shown in a lighter shade.

Networks generated by clustering disease signatures in the two cohorts highlighted the heterogeneity of clinical features observed in severe COVID-19. Such clustering enables the identification of disease signatures that co-occur in patient sub-groups who are likely to have similar symptoms, underlying conditions, or clinical outcomes. For example, hospitalized patients who developed ARDS were likely to be influenced by the features nearest to ARDS in the network such as older age, development of pneumonia, pulmonary hemorrhage, sepsis, and high mortality (Figure 3, Supplementary Figure 6).

### Disease Signatures Associated With Lower Levels of Serum Calcium and Lipids

In Cohort 2, features from five blood analytes (calcium, LDL, HDL, triglycerides, and leukocyte count) were available for patients. Hospitalized patients with severe COVID-19 were observed to be more likely to have lower serum calcium levels (<9.26 mg/dl), lower LDL levels (<78.23 mg/dl), lower HDL levels (<44.35 mg/dl), and higher levels of triglycerides (>206.20 mg/dl) when compared against the patients with mild disease (Supplementary Table 4). Both low and high levels of blood leukocyte count were observed in patients with severe COVID-19.

In Cohort 2 the PrecisionLife platform identified 18 disease signatures in 80 hospitalized patients with serum calcium values lower than 9.26 mg/dl (Supplementary Figure 7). Out of these, only four signatures were co-associated with the use of the dihydropyridines (calcium channel blockers) and proton-pump inhibitors which may have an effect on calcium homeostasis (27, 28). The hypocalcemia disease signatures were associated with COVID-19 symptoms such as pneumonia and respiratory failure, and comorbidities including diabetes, hypertension, and anemia. Two calcium disease signatures were found in 34 patients (42.5%), co-occurring with high mortality and hospital re-admission risk scores, which suggests that these patients had multiple underlying conditions. Another calcium disease signature in 33 (41.3%) patients was associated with low serum levels of HDL and pneumonia.

We also identified 45 disease signatures in 188 (32.4%) severe COVID-19 patients that were associated with comparatively low serum lipid (LDL, HDL, or triglyceride) levels (Supplementary Figures 8–10). Comorbidities such as hypertension, obesity, and cerebrovascular disease were found in these hypolipidemia signatures, which are not commonly co-associated in patients. We investigated whether the reduced lipid levels observed in these patients were caused by the use of statins. None of the disease signatures were associated with the feature indicating statin use by all associated cases. We found 12 hypolipidemia signatures where <10% of the patients were associated with any prescription records for statins within 90 days of the laboratory test result date, suggesting that these signatures were independent of statin use. Thus, dyslipidemia observed in many severe COVID-19 patients in Cohort 2 is not likely to represent an artifact of other comorbidities or medication use, but a consequential host response to SARS-CoV-2 infection which has been reported in many recent studies (29–31).

Mortality in the patients with either calcium or lipid disease signatures was not found to be significantly different. We were able to identify 15 disease signatures with lower levels of calcium and one signature with lower levels of cholesterol in this subcohort that were associated with at least 10 patients. The identification of calcium and lipid disease signatures in this subcohort strongly suggests that they reflect biochemical characteristics of patients with severe host response to COVID-19.

## Discussion

Pulmonary manifestations of COVID-19 such as respiratory failure and pneumonia were the most common symptoms in the two cohorts that were also prevalent in the combinatorial disease signatures identified by the PrecisionLife platform (Supplementary Figures 3, 5). Comorbidities such as hypertension, cardiovascular disease, chronic respiratory disease, and diabetes are known to be associated with COVID-19 risk from other studies (2–4), including our previous genetic study (18) in UK Biobank, were observed in hospitalized patients. These comorbidities co-occur with different COVID-19 symptoms, complications, medication use, and laboratory analyte values. This analysis enables us to gain useful insights into the likely associations between these clinical and phenotypic features that can improve the clinical management of patients.

A wide variety of severe COVID-19 manifestations, such as ARDS, sepsis, pericarditis, and thrombosis, were observed in the disease signatures representing patient sub-groups (2, 14, 24–26). This correlates with our previous genomic analysis on the UK Biobank COVID-19 cohort, which identified genes associated with some of these complications, including host pathogenic responses, inflammatory cytokine production, modulation of cardiac function, and endothelial cell function (19).

The use of medications such as proton pump inhibitors, dihydropyridines, and beta-adrenergic blockers was observed in seven disease signatures in Cohort 1 and 80 signatures in Cohort 2. Dihydropyridines (32, 33)and beta-adrenergic blockers (34, 35) have been associated with improved outcomes for COVID-19 patients and suggested as potential treatments, while proton pump inhibitors have been associated with adverse outcomes in several studies (36, 37). The incidence of the medications in the disease signatures could be either due to adverse effects caused by the medication resulting in a more severe COVID-19 response or it could reflect the comorbidities in patients for which they are generally prescribed. Using the available data, it was not possible for us to ascertain the specific association of these medications in our study with certainty.

In Cohort 2, all hypocalcemia (*n* = 18) disease signatures and hypolipidemia (*n* = 45) signatures were found to be associated with severe pulmonary manifestations of COVID-19 (Supplementary Figures 7–10). There is increasing evidence that calcium and lipid homeostasis plays an important role in the viral replication cycle and they have been suggested as biomarkers for increased COVID-19 severity (29–31, 38). It has been demonstrated that the calcium signaling pathway or calcium-dependent processes in host cells are often perturbed by viral proteins that can bind calcium and/or calcium-binding protein domains, allowing them to modulate the host cellular machinery for viral replication, assembly, and release (39, 40). The mechanism of calcium regulation is not fully understood, as some viruses are known to increase intracellular calcium levels while others are known to have a dynamic control based on the phase of infection (41). However, the SARS-CoV E protein has been shown to form protein-lipid channels that transport calcium ions, activating the NLRP3 inflammasome and increasing systemic inflammation via IL-1β (42).

Lower lipid levels have been reported in severe COVID-19 patients in many studies with a correlation observed between reduced lipid levels and disease severity (43–45). Many viruses, including SARS-CoV and MERS-CoV, can modulate lipid synthesis and signaling in host cells to divert cellular lipids to viral replication and exocytosis, facilitating the invasion of other host cells (46, 47). It has been suggested that the decrease in cellular cholesterol levels following SARS-CoV-2 infection leads to disruption of the signaling hub for inflammation and cholesterol metabolism, resulting in the dysregulation of cholesterol biosynthesis, inflammatory cytokine release, and vascular homeostasis (48, 49).

Regulation of cholesterol biosynthesis has been shown to be associated with six genes identified by a genome-scale CRISPR knockout screen that reduced SARS-Cov-2 infection in human alveolar basal epithelial carcinoma cells (50). The study also demonstrated that the use of dihydropyridines results in increased resistance to SARS-Cov-2 infection (50). Another study hypothesized that elevated unsaturated fatty acids in SARS-CoV-2 infected host cells bind calcium, resulting in hypocalcemia and triggering the production of pro-inflammatory mediators and cytokine storm induction (51, 52).

We found seven disease signatures in this study where lower levels of LDL were found co-occurring with lower levels of serum calcium, leukocyte count, or HDL. These signatures may be attributed to similar mechanisms linking calcium and lipid signaling where changes in cellular lipid levels during inflammation and infection (53) affects calcium signaling in host cells (54–56).

Retrospective analysis of the clinical histories of the hospitalized patients with lower calcium and lipid signatures was performed to identify whether the laboratory analyte values may be affected by other medical conditions. We found that 50% of cases represented by disease signatures featuring lower levels of calcium were reported to have vitamin D deficiency which is important for calcium homeostasis in both physiological and disease states (57). More than 25% of people above the age of 65 were vitamin D deficient. This suggests that the changes in calcium levels in some patients may be linked to vitamin D deficiency in severe COVID-19 (57, 58), which has also been associated with severe illness and which was found in eight disease signatures in Cohort 2. A recent study reported that lower serum calcium levels have been found to be associated with COVID-19 patients with pneumonia independent of vitamin D deficiency (59). This finding is consistent with our findings that a sub-group (50%) of patients with low serum calcium values were not reported with vitamin D deficiency. It is likely that in these patients, the changes in lipid levels following COVID-19 infection (53) affects the serum calcium levels (54–56), similar to the patients who had disease signatures that were combinations of lower serum calcium, leukocyte, and HDL levels.

Our previous analysis on the UK Biobank COVID-19 cohort (19) identified 16 calcium-binding/signaling genes and six genes relating to lipid droplet biology and correlated with serum lipid levels and coronary artery disease. In conjunction with the findings of this study, this adds further support to the role of calcium and lipid signaling in relation to viral pathogenesis and severe host response to COVID-19. To fully understand the role of calcium and lipid homeostasis in COVID-19, analysis of patient datasets that combine genetic, clinical, and hospital laboratory test data will be necessary.

### Limitations of the Study

This study was limited by the completeness of data for features relevant to analyzing differential host response to COVID-19. Information on the onset of disease or symptoms, the clinical phase of disease, viral load, oxygen saturation, breathing rate, body mass index, and physiological measurements or biomarker levels during hospitalization was not consistently available. We used hospitalization status associated with a primary diagnosis of COVID-19 as a surrogate for severe COVID-19 patients. Mortality and diagnoses linked to clinical progression of COVID-19 were used to estimate the relative severity of disease among hospitalized patients.

The comorbidities, diagnoses, medications, and laboratory test results were derived from medical claims, pharmacy claims, and in-patient admission records. Since claims data are generated for reimbursement and administrative purposes rather than scientific research, the records may be missing information and there is potential for variability in their collection. Data sparsity of the available patient records was reflected in the low penetrance of many disease signatures. As more patient data becomes available, the disease signatures will become more predictive, enabling higher resolution patient stratification.

## Conclusion

The PrecisionLife platform identified and analyzed 836 combinatorial disease signatures in two COVID-19 cohorts (Cohort 1 = 255, Cohort 2 = 531) associated with increased risk of hospitalization from COVID-19. These disease signatures were found to capture different symptomatic presentations of COVID-19, complications arising from the clinical progression of the disease, and underlying disease conditions that could be either associated with severe host response to COVID-19 or were indicative of conditions associated with older age or frailty.

In Cohort 2, we found 45 disease signatures that were associated with lower levels of serum calcium, LDL, HDL, and triglycerides in 188 (32.35%) hospitalized patients. This suggests that lower levels of calcium and cholesterol are biochemical characteristics associated with severe COVID-19 patients, which may also add further support to the role of calcium signaling and lipid dysregulation in SARS-CoV-2 pathogenesis.

These findings are consistent with the insights generated by multiple studies in different COVID-19 patient populations. This also validates our findings from our previous genomics study (19) on severe COVID-19 patients in UK Biobank (20) where we identified 16 risk-associated genes that had calcium-binding domains or were involved in calcium signaling and six genes linked to lipid droplet biology associated with serum lipid levels.

This study along with our previous genomic study (19) demonstrates that a combinatorial analysis approach is able to identify related groups of clinical and phenotypic features from both genomic and phenotypic data that are associated with the risk of developing severe forms of COVID-19. This enables us to gain unique insights into the non-linear combinatorial feature associations to a clinical phenotype in patient sub-groups, that is not detected by standard data analysis approaches. With the availability of more data, the combinatorial output of the analytical platform would be greatly enhanced and the insights derived from them would allow for the identification of targeted approaches to patient care.

This analysis also validates the association of calcium and lipid homeostasis with severe COVID-19 reported by our previous study, using real-world data in an independent cohort. We will extend these analyses in future to larger patient datasets that have both genetic and phenotypic data to fully ascertain the differences between mild and severe host responses to COVID-19 and the mechanism of calcium and lipid signaling in SARS-Cov-2 pathogenesis.

## Data Availability Statement

The data analyzed in this study is subject to the following licenses/restrictions: the data analyzed in this study was obtained from UnitedHealth Group Clinical Discovery Portal. The data are proprietary and are not available for public use but, under certain conditions, may be made available to editors and their approved auditors under a data-use agreement to confirm the findings of the current study. Requests to access these datasets should be directed to Scott Schneweis, schneweis@uhg.com.

## Ethics Statement

Ethical review and approval was not required for the study on human participants in accordance with the local legislation and institutional requirements. Written informed consent for participation was not required for this study in accordance with the national legislation and the institutional requirements.

## Author Contributions

SG conceived and supervised the study. MP and SD performed the studies and analyzed the data. SD wrote the manuscript. KT contributed to the study design, analysis of disease signatures, and manuscript. VB and MS contributed to the study design and manuscript. GM developed the core technology in PrecisionLife's platform. TH and KTHT contributed to the study design and coordinated access to the COVID-19 Data Suite through the UHG Clinical Discovery Portal. All authors contributed to the study and approved the final version of the manuscript.

## Conflict of Interest

SD, MP, KT, VB, GM, MS, and SG were employed by company PrecisionLife Ltd. TH and KTHT were employed by company OptumLabs at UnitedHealth Group.
