# Supplementary material for: Combinatorial Analysis of Phenotypic and Clinical Risk Factors Associated With Hospitalized COVID-19 Patients
Source: Front Digit Health. 2021 Jul 8;3:660809. doi: 10.3389/fdgth.2021.660809 (PMC8521999; doi:10.3389/fdgth.2021.660809)
Supplement: Supplementary file 1 [file Data_Sheet_1.pdf]

Supplementary Material for
Combinatorial analysis of phenotypic and clinical risk factors associated with hospitalized COVID-19 patients

Sayoni Das[1,†], Matthew Pearson[1,†], Krystyna Taylor[1], Veronique Bouchet[1], Gert Lykke Møller[1], Taryn O. Hall[2], Mark Strivens[1], Kathy T. H. Tzeng[2], Steve Gardner[1,*]

[1] PrecisionLife Ltd., Oxford, United Kingdom
[2] OptumLabs at UnitedHealth Group, Minnetonka, MN, United States

*** Correspondence:** Steve Gardner steve@precisionlife.com

† These authors have contributed equally to this work.

1 Mining Terminology and Example

The overall process of mining, validation and scoring is shown below.

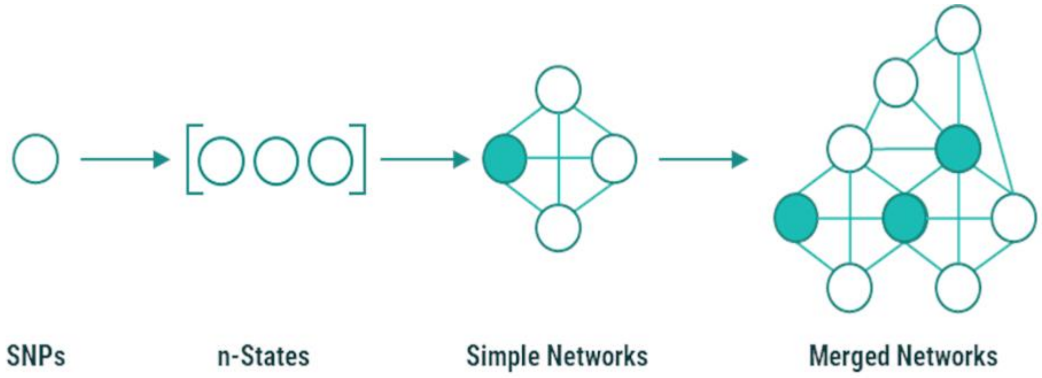

Supplementary Figure 1. Stages of the mining, scoring and analysis process in PrecisionLife's combinatorial analytics platform.

2 Supplementary Data

Supplementary Table 1. COVID-19 positive patient records available in the UnitedHealth Group (UHG) COVID-19 Data Suite.

	**Hospitalized patients**	**Non-hospitalized patients**
COVID-19 positive patients	37,027	219,271
COVID-19 patients with any clinical data since 2019	19,244	28,408
Cohort 1 - COVID-19 patients with clinical data who were not enrolled in Medicare Special Needs Plan	3,183	6,310
Cohort 2 - Cohort 1 patients with additional laboratory data for 5 lab analytes	581	1,000

Supplementary Table 2. Features used for hospitalization risk studies.

**Feature group**	**Data source**	**Description**	**Feature count**
Sex	Member information	1 indicates Male, 0 indicates Female, 3 indicates missing data	1
Race	Member information	1 indicates non-Caucasian, 0 indicates Caucasian and 3 indicates missing data.	1
Co-morbidity flags based on Co-morbidity Index	Co-morbidity scores table derived by UHG from medical claims	1 indicates incidence of at least one claim associated with co-morbidity flag since 2019 and 0 indicates its absence.	32
ICD-10-CM level 2 codes	Medical claim	ICD-10-CM level 2 codes (excluding those used as co-morbidity flags) occurring in at least one medical claim since 2019 are indicated by 1 and their absence by 0.	1,018
Medications	Pharmacy claim	1 indicates incidence of at least one claim associated with the therapeutic class of medication since 2019 and 0 indicates its absence.	282
Age	Member information	Values above 0.5 SD were assigned 1 and those below 0.5 SD were assigned 0. Values in between were considered missing and assigned the value 3.	1
Risk scores based on Co-morbidity index		Same as age	4
Lab analytes	Lab data	Same as age	5

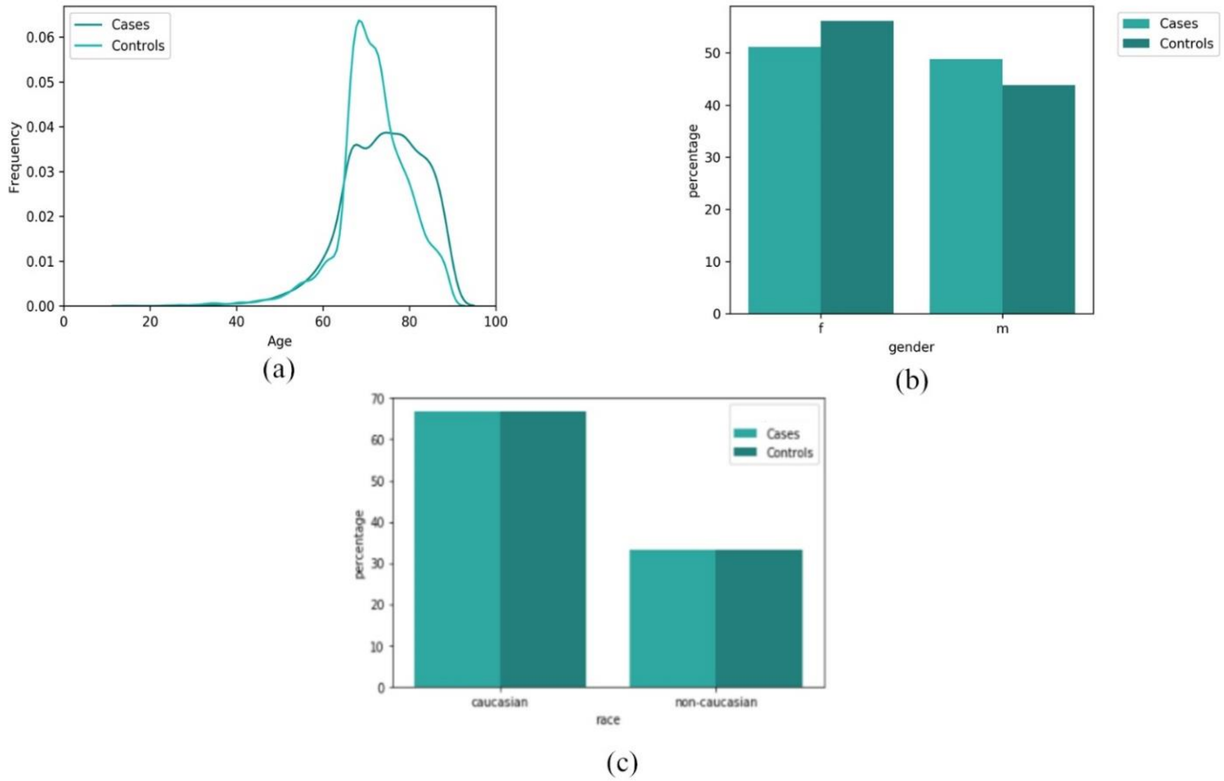

Supplementary Figure 2. Distribution of (a) age, (b) gender and (c) race for the full hospitalization risk cohort (Cohort 1, n=9,493 comprising of 3,183 cases and 6,310 controls).

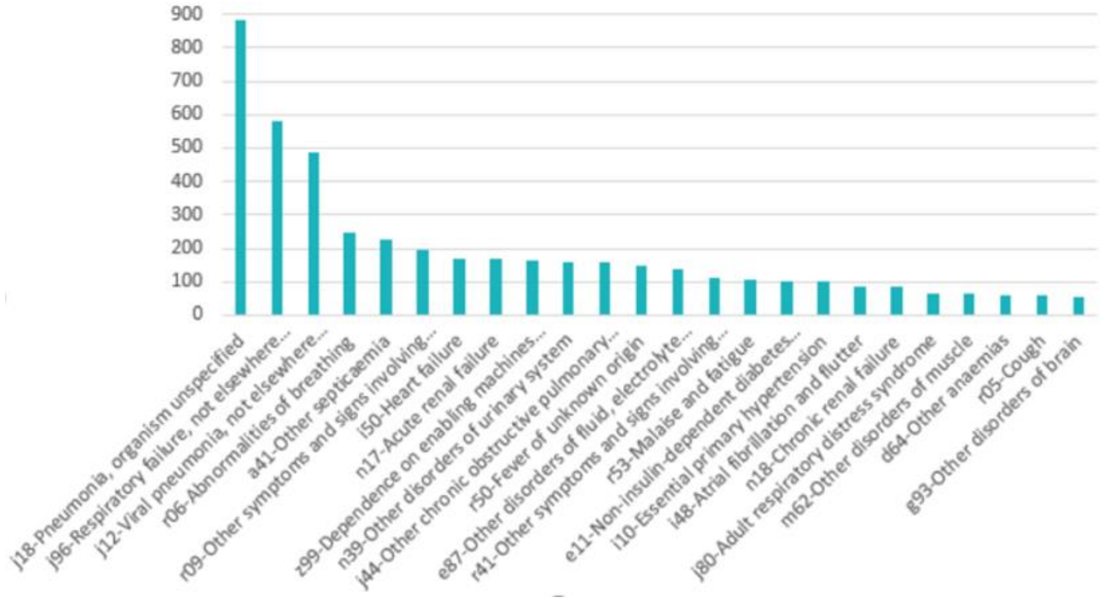

Supplementary Figure 3. Most frequently associated diagnoses associated with COVID-19 patients during their hospitalization in the full hospitalization risk cohort (Cohort 1).

Supplementary Table 3. Summary of combinatorial analysis results for the Hospitalization risk studies.

	**Cohort 1**	**Cohort 2**
**Cases**	3,183 (males: 1,549, females: 1,634)	581 (males: 295, females: 286)
**Controls**	6,310 (males: 2,758, females: 3,538)	1,000 (males: 438, females: 560)
**Features**	1,339	1,344
**False Discovery Rate (FDR)**	0.01	0.01
**Random Permutations**	2,500	2,500
**Disease Signatures (N-states)**	1,147	32,242
**Penetrance**	67.29%	82.61%
**Filtered Disease Signatures**	255	531
**Penetrance of Filtered Disease Signatures**	54.97%	59.72%
**Critical features**	166	41

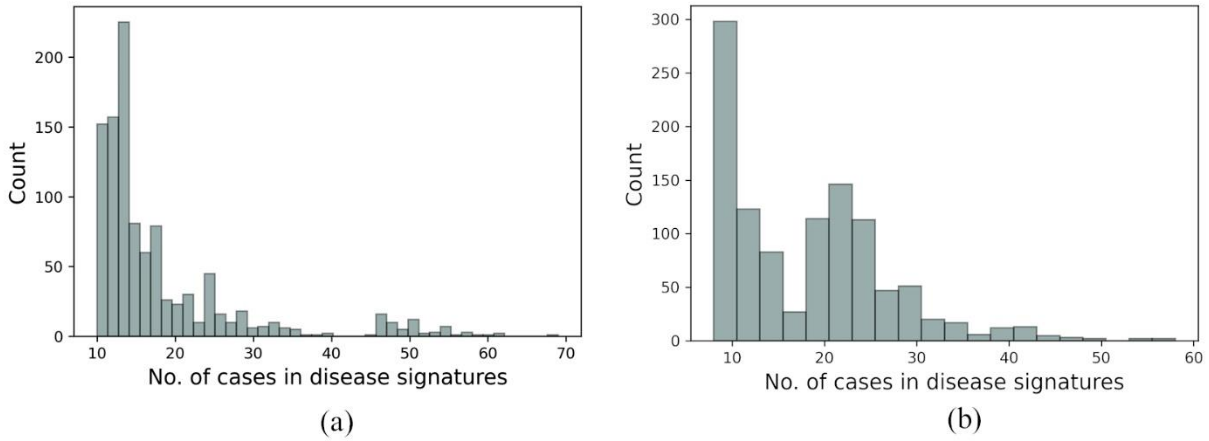

Supplementary Figure 4. Distribution of cases in COVID-19 disease signatures (excluding signatures that had any feature indicating absence of a disease diagnosis, symptom or medication) identified in (a) Cohort 1) and (b) Cohort 2.

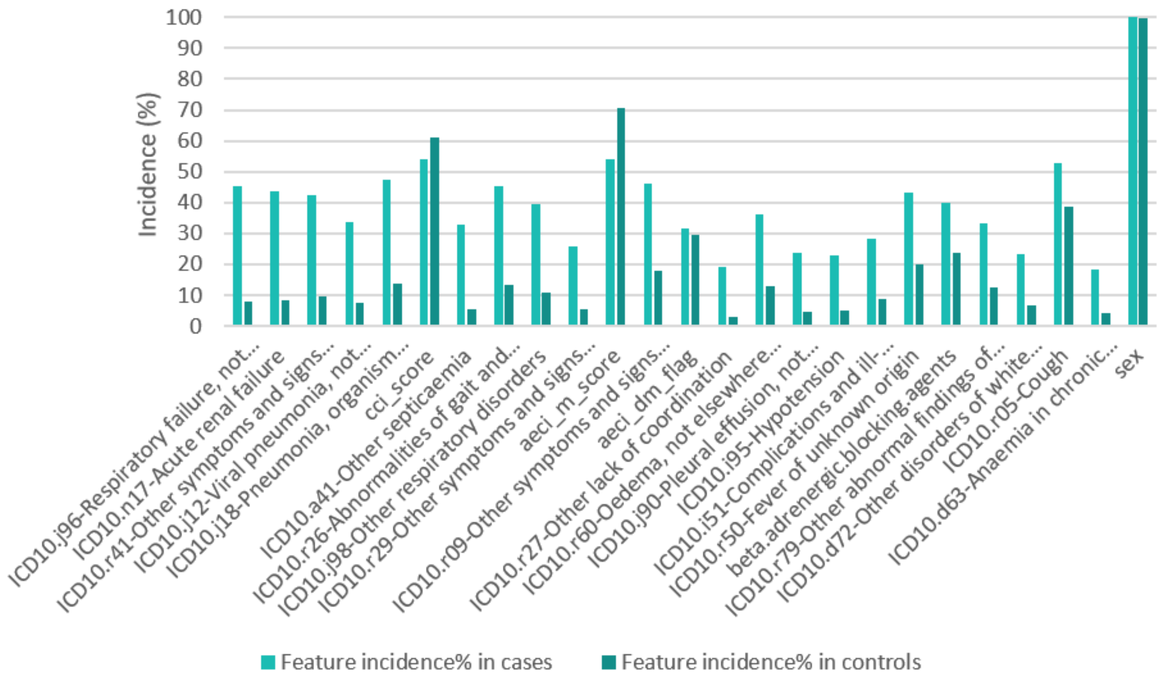

(a)

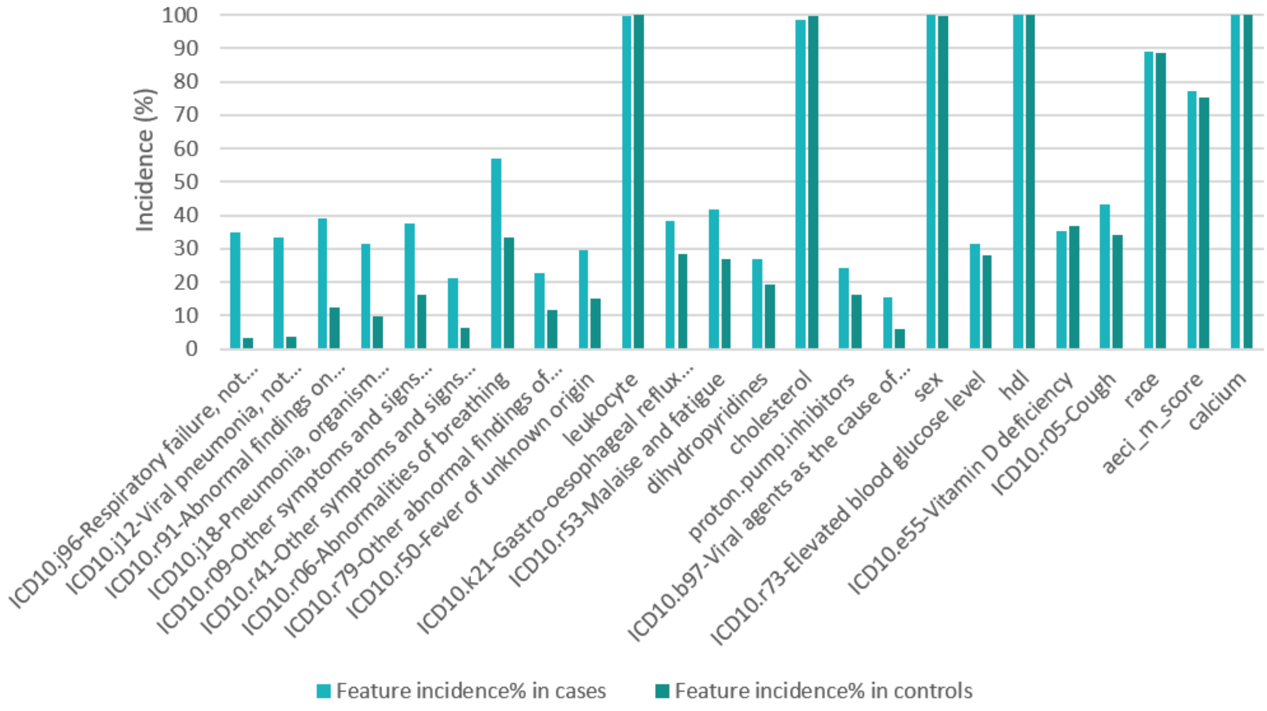

(b)

Supplementary Figure 5. Incidence of the top Random Forest **(**RF) scored critical features in cases and controls for (a) Cohort 1 and (b) Cohort 2.

Supplementary Table 4. Incidence of features derived from laboratory test results of patients in the cohort for the hospitalized risk study using lab data. *p-values* were calculated to assess the association of each feature in the two COVID-19 hospitalized risk cohorts using two-sided Fisher's exact tests.

**Lab analyte (normal range)**	**Lab analyte feature**	**Lab feature definition**	**Cases (n=581)**	**Controls (n=1000)**	**Two-sided *p-value***	**One-sided *p-value* (lesser)**	**Disease signatures**
Calcium	Calcium:0	< 9.26 mg/dl in serum plasma	289	770	0.0047	0.0024	18
(8.6-10 mg/dl)	Calcium:1	> 9.71 mg/dl in serum plasma	231	823	0.0047	0.998	4
LDL	LDL:0	< 78.23 mg/dl in serum plasma	345	858	4.5e-09	2.8e-09	26
(100 -129 mg/dl)	LDL:1	>114.37 mg/dl in serum plasma	224	984	4.5e-09	1	0
HDL	HDL:0	< 44.35 mg/dl in serum plasma	386	971	8.4e-10	4.2e-10	15
(40-60 mg/dl)	HDL:1	> 66.16 mg/dl in serum plasma	188	871	8.4e-10	1	4
Triglycerides	Triglycer:0	< 73.05 mg/dl in serum plasma	122	553	0.0002	0.9999	6
(100-150 mg/dl)	Triglycer:1	> 206.20 mg/dl in serum plasma	150	404	0.0002	0.00105	0
Leukocyte count	Leukocyte: 0	< 5.28 k/mm3 in blood	194	831	9.2e-09	3.9e-89	8
(5-10 k/mm^3)	Leukocyte: 1	> 8.17 k/mm3 in blood	240	542	9.2e-09	4.79e-07	9

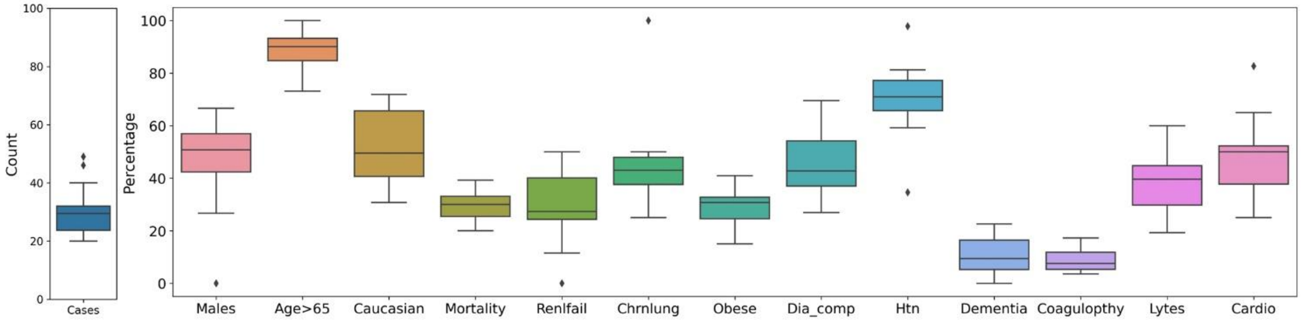

Supplementary Figure 6. Clinical characteristics of the disease signatures of hospitalized patients in Cohort 1 who had developed ARDS. Each boxplot captures the distribution of the incidence of a clinical feature in all ARDS disease signatures.

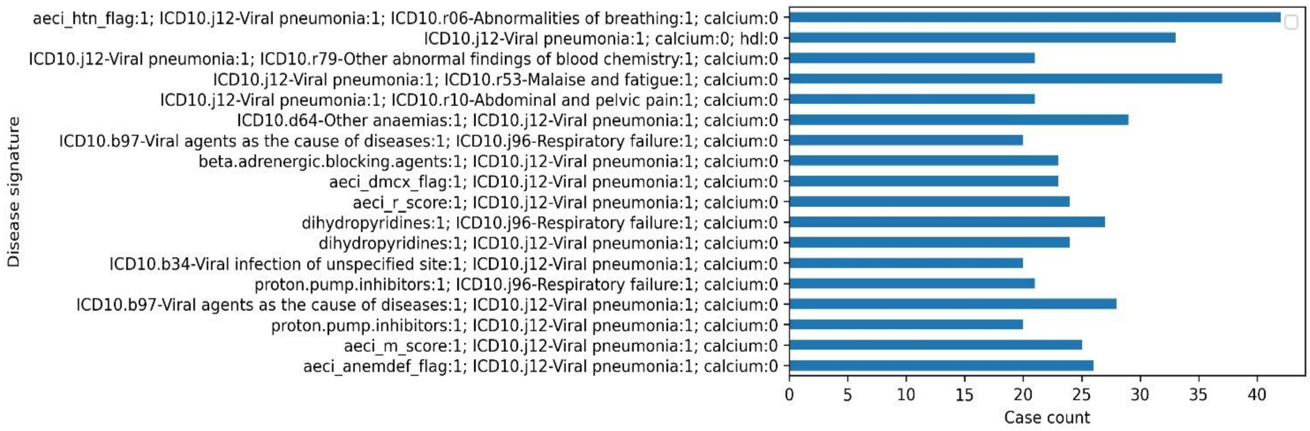

Supplementary Figure 7. Disease signatures with serum calcium levels below 9.11 mg/dl reported in Cohort 2.

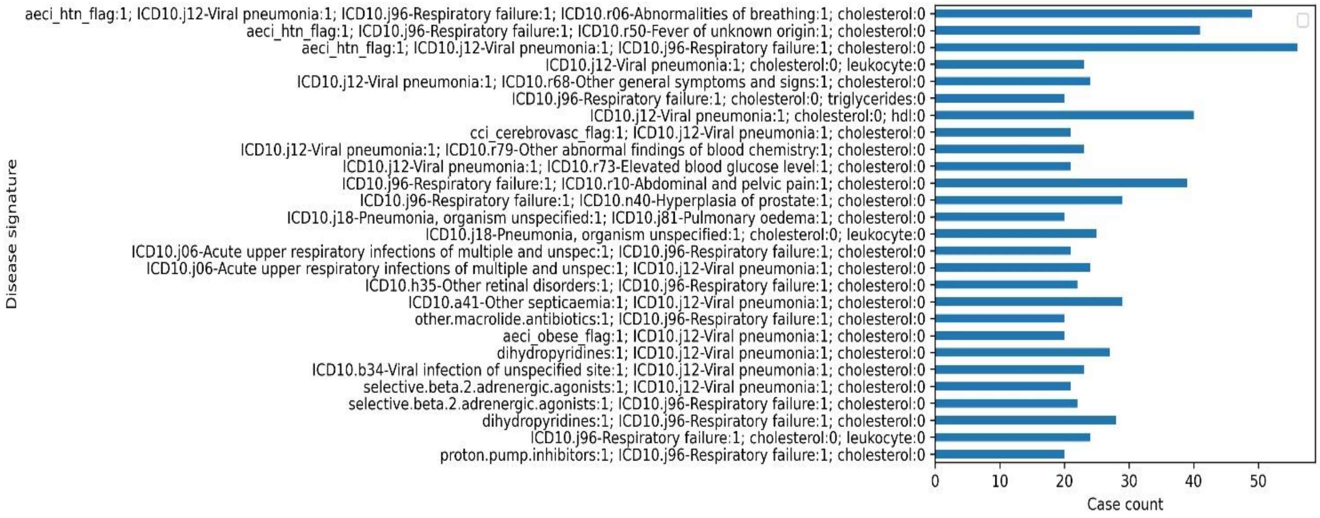

Supplementary Figure 8. Disease signatures with serum cholesterol (LDL) levels below 78.23 mg/dl reported in Cohort 2.

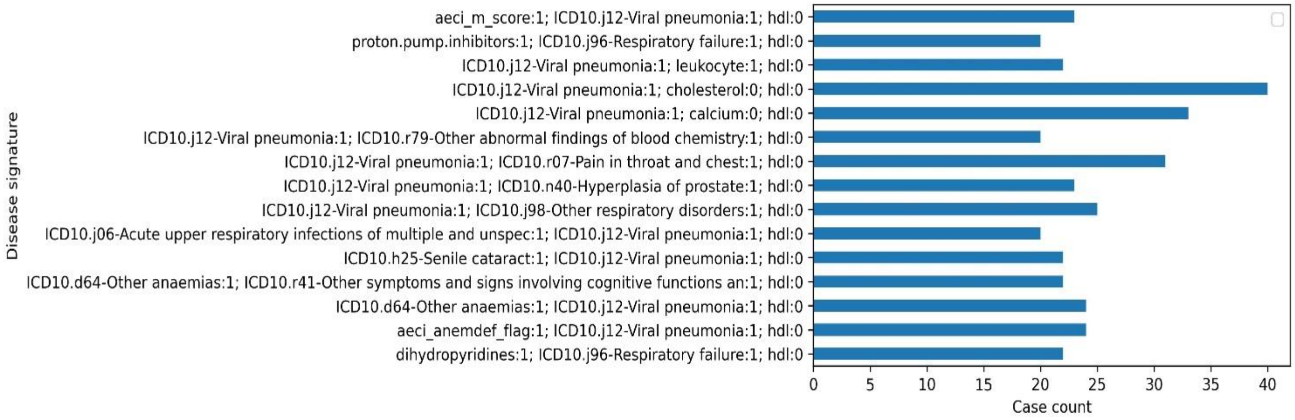

Supplementary Figure 9. Disease signatures with serum HDL levels below 44.35 mg/dl reported in Cohort 2.

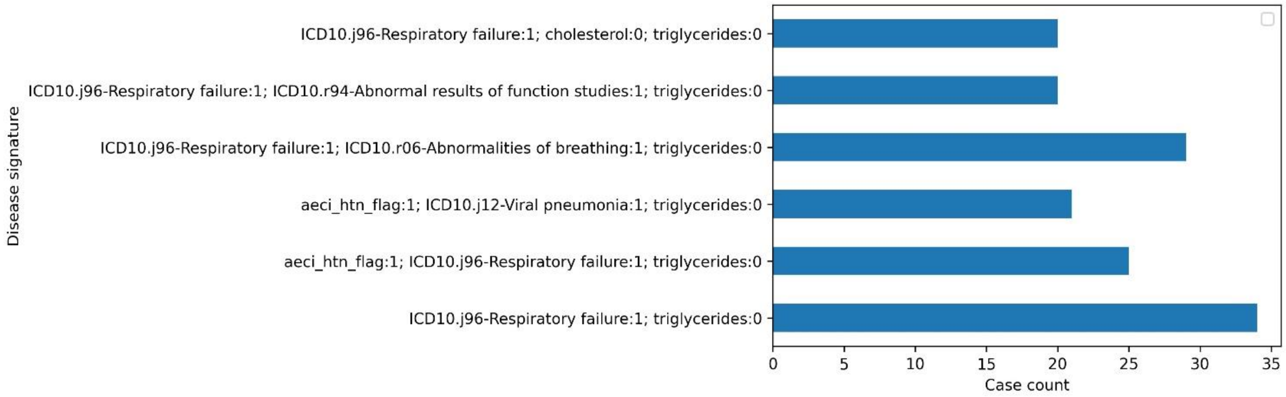

Supplementary Figure 10. Disease signatures with serum triglyceride levels below 73.05 mg/dl reported in Cohort 2.
